# The Impact of X‐ray Damage in Chemical Crystalline Materials and New Opportunities for Small Molecule Serial Crystallography

**DOI:** 10.1002/chem.202501711

**Published:** 2025-07-29

**Authors:** Sam G. Lewis, Mark R. Warren, Lauren E. Hatcher

**Affiliations:** ^1^ School of Chemistry Cardiff University, Main Building Park Place, CF10 3AT Cardiff UK; ^2^ Diamond Light Source Harwell Science and Innovation Campus Fermi Avenue, OX11 0DE Oxfordshire Didcot UK

**Keywords:** phase transitions, radiation damage, serial crystallography, solid‐state reactions, X‐ray diffraction

## Abstract

In line with the dramatic and continuing improvements in X‐ray generation technologies for laboratory and accelerator sources around the world, reports of X‐ray‐induced processes in chemical crystalline materials are on the rise. These observations are challenging the traditional viewpoint that radiation damage is only of significant concern for protein structure determinations, encouraging small molecule crystallographers to identify and address X‐ray‐induced effects in a broadening range of susceptible materials. This review explores the recent investigations into X‐ray damage and X‐ray‐induced transformations in chemical crystalline materials and discusses state‐of‐the‐art methodologies to quantify and mitigate the effects of the X‐ray probe on single‐crystal and microcrystalline powder samples.

## Graphical abstract

The impact of X‐ray damage in chemical crystals was minimised by the introduction of area detectors in the late 20^th^‐century. Now, ever‐brighter X‐ray sources are re‐establishing X‐ray damage as a key challenge. This article reviews recent observations of X‐ray‐induced processes in chemical samples and discusses state‐of‐the‐art methodologies for their mitigation.

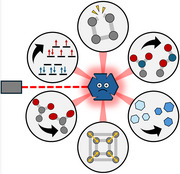

## 1 Introduction

Since Max von Laue and coworkers first observed the X‐ray diffractive properties of crystalline materials in 1912, there have been remarkable technological developments across the field. Among these advances is the rapid increase in the brilliance of the X‐ray sources available to the modern‐day chemical crystallographer. Commercial laboratory diffractometers are now routinely equipped with high‐flux sources, including microfocus rotating anodes and even groundbreaking new liquid metal jet X‐ray sources that offer intensities of > 10^12^ X‐rays sec^−1^ mm^−2^.^[^ ^1^ ^]^ Beyond this, upgrade projects at synchrotrons around the world are delivering increases in brilliance and output X‐ray flux of several orders of magnitude compared to their predecessor machines.^[^ ^2^, ^3^, ^4^ ^]^ Simultaneously, growing numbers of X‐ray free electron lasers (XFELs) are coming online that have a peak brilliance approaching 10^10^ times that of third‐generation (pre‐upgrade) synchrotron facilities.^[^ ^5^ ^]^ These rapid and unprecedented improvements now permit the study of ever smaller and weakly diffracting crystals and enable increasingly complex in situ experiments to be run. While these developments are undoubtedly exciting for the field of chemical (or “small molecule”) crystallography, the advent of ever brighter sources is challenging a long‐held assumption: that X‐rays are an innocent probe whose interaction with the sample is negligible and can thus be disregarded. However, in recent years there have been an increasing number of studies reporting X‐ray‐induced effects on small molecule crystal systems, including X‐ray damage and X‐ray‐induced transformations, indicating this assumption is no longer valid. As the availability of ultrabright sources continues to rise, efforts to understand, mitigate, and even harness these consequential effects are increasingly important to ensure the capabilities at new facilities are fully realized.

The phenomenon of X‐ray‐induced damage has been considerably better studied in proteins by the macromolecular crystallography (MX) community than for small molecules, with studies of X‐ray damage first reported in the early 1960s.^[^ ^6^ ^]^ As such, there are a significant number of reviews and individual reports covering this area in detail, and the authors refer the reader to the many excellent texts for a comprehensive overview.^[^ ^7^ ^]^ In brief: X‐rays interact with a crystal via three core mechanisms. Thompson (coherent, or elastic) scattering occurs when an X‐ray photon is scattered with no energy losses and is the mechanism on which the diffraction experiment is based. The remaining interactions, Compton (incoherent) scattering and photoelectric absorption, involve energy loss from the X‐ray photon into the sample and thus both contribute to radiation damage.^[^ ^7i^ ^]^ X‐ray damage processes can be categorized as either primary, secondary, or tertiary. Primary damage occurs as a result of initial X‐ray absorption and the ejection of a photoelectron, leading to problems such as atomic ionization and radiolysis of crystallized water to form hydroxyl radicals. Secondary damage is caused by the products of primary processes, for example, radicals interacting with susceptible protein residues. At the extreme, tertiary damage refers to changes in the wider crystal structure on the accumulation of radiation‐induced effects, ultimately resulting in the loss of crystallinity. X‐ray absorption by a crystal is measured in terms of the radiation dose, which has the SI unit of Gray (Gy, or J kg^−1^).^[^ ^8^ ^]^ Research has shown that the dose limit can be significantly increased by cryocooling, and the accepted dose limit for protein crystals at 100 K is 30 MGy.^[^ ^7c^ ^]^ Above this threshold, a significant decrease in the diffraction quality is observed with peak intensities falling by > 70% of their original values. As specific damage in the crystal (primary, secondary, or tertiary) starts to occur at progressively high doses, it is useful to quantify the dose received and work in this area has been led by Garman and coworkers. Their dedicated software, *RADDOSE‐3D*,^[^ ^9^ ^]^ can be used to estimate the diffraction‐weighted dose absorbed by a crystal given a set of sample (e.g., crystal size, unit cell parameters, and elemental composition) and X‐ray (e.g., energy, flux, beam size, and profile) related variables. *RADDOSE‐3D* has recently been updated to work with chemical systems,^[^ ^10^ ^]^ to consider the use of capillaries for small‐angle X‐ray scattering (SAXS) experiments,^[^ ^11^ ^]^ and to assess radiation damage from electron beams.^[^ ^12^ ^]^ Hence, the tools to quantify radiation damage across a broad range of materials and experiments are now available, providing the opportunity to quantify the increasingly common incidence of X‐ray damage in chemical crystals and deliver more accurate structure determinations in the face of these effects.

Alongside quantification, there exist a number of strategies to mitigate radiation‐induced effects before significant damage can occur. For example, the use of small microcrystals can help alleviate radiation damage via the phenomenon of photoelectron escape.^[^ ^13^ ^]^ Where a crystal is small enough (≤10 µm in a single dimension), a considerable proportion of the primary photoelectrons escape the crystal before they can propagate further damage events. Another mitigation approach is to collect data quickly enough to “outrun” damage processes, often through multi‐crystal methodologies such as serial crystallography (SX).^[^ ^14^ ^]^ In SX data collections, a stream of microcrystals is delivered into the beam and, typically, only a single diffraction image is collected on each crystal. To produce a complete dataset for structure determination, the diffraction images from thousands of individual microcrystals are then merged and scaled together. This “single shot” approach effectively spreads the total X‐ray dose across a large number of crystals, allowing data to be collected ahead of significant damage accumulation in any one sample. SX was initially developed for “diffract‐before‐destroy” serial femtosecond crystallography (SFX) experiments,^[^ ^14a^ ^]^ a necessity as just a single exposure from the free electron laser (FEL) frequently destroys a crystal. More recently, serial synchrotron crystallography (SSX) methods have been developed, allowing researchers to capitalize on the enhanced flux available at upgraded facilities and to explore increasingly complex in situ measurements.^[^ ^14^, ^15^ ^]^ SX has again been pioneered by the MX community. For chemical samples, the concept of spreading X‐ray dose across multiple crystals is now gaining traction for both synchrotron and XFEL studies. In this article, we aim to review the reports of X‐ray‐induced changes in chemical crystals, exploring the extent and trajectory of these observations and their role in motivating the development of new experimental protocols. We also summarize the recent exploratory work to adapt SX for small molecule samples and provide an outlook for the wider use of small molecule SX (smSX) in the near future.

## 2 X‐Ray‐Induced Radiation Damage & Induced Structural Changes

Despite being vastly under‐researched in comparison to proteins, X‐ray‐induced radiation damage in chemical crystals is not a new phenomenon, and initial investigations date back to the late 1970s and early 1980s.^[^ ^7^, ^16^ ^]^ However, the widespread introduction of charge‐coupled device (CCD) detectors in the mid‐1990s significantly reduced X‐ray diffraction collection times.^[^ ^17^ ^]^ As a result, the reports of radiation damage subsided.^[^ ^18^ ^]^ More recently, however, studies detailing X‐ray‐induced processes are once again on the rise, being observed across an increasingly broad range of materials and, in many cases, accompanied by correlated changes in the materials’ physical properties. This section provides an overview of the recent literature inspired by the renewed interest in X‐ray‐induced phenomena and reflects on the growing opinion that X‐ray damage is not solely problematic but instead presents a new opportunity to manipulate and tune the properties of radiation‐sensitive solids.^[^ ^19^ ^]^

### 2.1 Sample Decomposition and Disorder

**Figure 1:**
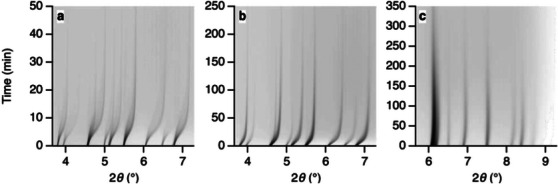
X‐ray powder diffraction intensities, plotted as a function of X‐ray exposure time and measured at ambient temperature, for the series of zeolitic imidazolite frameworks (ZIFs) studied by Widmer et al.: a) ZIF‐4, b) ZIF‐62, and c) ZIF‐zni. [Reprinted with permission from Widmer et al*., Physical Chemistry Chemical Physics* 2019, 21, 12 389–12395. Copyright 2019 Royal Society of Chemistry.]^[^ ^20^ ^].^

In 2019, Widmer et al. reported the amorphization of a series of zeolitic imidazolite frameworks (ZIFs): ZIF‐4, ZIF‐62, and ZIF‐zni, by repeated X‐ray irradiation during powder X‐ray diffraction (PXRD) studies conducted on the Materials Science beamline at the Swiss Light Source (SLS).^[^ ^20^ ^]^ While the amorphization of such metal‐organic frameworks (MOFs) is of scientific interest for their increased defect concentrations and increased energy states,^[^ ^21^ ^]^ this phenomenon has significant implications for structure determination of MOFs by diffraction methods.^[^ ^22^ ^]^ At ambient temperature, the time taken for the diffraction intensity recorded from ZIF‐4, ZIF‐62, and ZIF‐zni samples to drop to 50% of its original value was found to be 6, 14, and 81 minutes of cumulative X‐ray irradiation, respectively, which was accompanied by peak broadening and an initial decrease in unit cell dimensions before leveling out upon further irradiation (Figure 1). This amorphization process is understood to arise from the accumulation of local defects over time, eventually leading to structural collapse of the crystalline framework. The basis for X‐ray‐induced damage is thought to be photooxidation at the Zn(II) centers, as subsequent recrystallization of the amorphized ZIF‐4 leads to the formation of the ZIF‐zni phase upon heating, confirming that the organic linker remains intact. The authors hypothesize that this observation correlates with the X‐ray energy used: at SLS an energy of 20 keV was applied, whilst a comparative study at Diamond Light Source (DLS) using 29 keV resulted in no significant amorphization. Interestingly, in a previous study conducted by the authors on similar materials at 20 keV, and prior to upgrades at the SLS, no amorphization was observed.^[^ ^23^ ^]^ This indicates that the increased flux resulting from SLS undulator upgrades may be responsible for the damage seen, highlighting the impact that ongoing upgrade projects may have for routine diffraction experiments at facilities around the globe.

**Figure 2:**
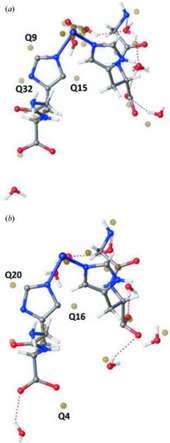
Single‐crystal X‐ray structures of *catena*‐bis(*μ*_2_‐glycyl‐histidinato‐*N,N',O*)nickel(II) heptahydrate during systematic X‐ray data collections performed by Christensen et al. to quantify the structure changes in the complex as a function of total X‐ray dose: a) structure determined from the first data collection after a total accumulated dose of 0.63 MGy, b) structure determined from the twelfth data collection after a total accumulated dose of 7.56 MGy. The largest positive Fourier electron density difference peaks (known as “q‐peaks”) are shown in both structures as unconnected gold spheres, and the difference in the distribution of these peaks about the left‐hand carboxylate group (see Q4 in b) illustrates the likelihood of an X‐ray‐induced COO^−^ to COOH transformation. [Reprinted with permission from Christensen et al*., IUCrJ* 2019, 6, 703–713. Copyright 2019 International Union of Crystallography.]^[10].^

In the same year, seminal work by Christensen, Garman, Coles et al. studied the heavily hydrated and moderately X‐ray susceptible complex *catena*‐bis(*μ*_2_‐glycyl‐histidinato‐*N,N',O*)nickel(II) heptahydrate, as shown in Figure 2.^[^ ^10^ ^]^ Systematic data collections were performed across a range of temperatures from 30 to 120 K, and at different beam attenuations on the small molecule crystallography beamline I19 at DLS.^[^ ^24^ ^]^ These measurements were used to quantify the influence of experiment parameters for controlling X‐ray damage. At all temperatures, upon repeated single crystal X‐ray diffraction (SCXRD) data collections, a decrease in the diffraction limit and the normalized summed diffracted intensity across the dataset was observed as a function of absorbed dose, although lowering the temperature reduced the rate of damage progression. At 100 K, the dose at which the diffraction intensity dropped to ca. half of its starting value (*D_1/2_*, a measure of dose limit) was determined to be just 4.4 MGy, which is considerably lower than the limiting dose for many protein crystals.^[^ ^25^ ^]^ However, this is in part attributed to the fact that the small molecule crystal displays much higher initial diffraction resolution compared to that seen for typical protein samples. Although the authors conclude that the observed changes in diffraction intensities provide the best metrics to monitor X‐ray damage in this nickel(II) complex, they also explore the impact on the final crystal structure model. While residual factors corresponding to the measured data (e.g., *R*_merge_, which quantifies the agreement between symmetry‐related measurements on the same Bragg peak) were proven to double during the experiment, residual factors for the structure model (e.g., the commonly‐quoted *R*_1_) increased by a lesser amount. This is interesting and reflects that care should always be taken to consider the limitations of residuals, such as *R*_1_, as their values can mask fundamental issues with the raw data, including those caused by X‐ray damage. Disorder in the model also increased with cumulative X‐ray dose, making interpretation of structural features increasingly difficult. This was particularly evident around the crystallized water molecules, which become steadily more disordered with each consecutive data collection. By the final experiment, corresponding to a total dose of 7.56 MGy, the carboxylic acid moiety surrounded by disordered waters (right‐hand side of Figure 2b) could no longer be modeled satisfactorily. Furthermore, changes around the dipeptide ligand also indicate the potential for radiation‐induced transformation in this moiety. The positions of residual positive Fourier electron density difference peaks (commonly referred to as “Q‐peaks,” e.g. Q4 in Figure 2b) could indicate an X‐ray‐induced COO^−^ to COOH transformation at high dose levels; however, care should be taken to consider the significant decrease in the data quality with successive data collections. In summary, this article presented a systematic and quantitative approach to understanding radiation damage in a molecular crystal, challenging the small molecule crystallography community to revisit these phenomena in their own datasets and providing new tools and protocols for study.

### 2.2 Spin‐Crossover

The phenomenon of spin‐crossover (SCO) has been of great interest to the scientific community since the first reports of the magnetic properties in a series of Fe(dialkyldithiocarbamate) complexes by Cambi et al. in 1931.^[^ ^26^ ^]^ SCO refers to the ability of certain transition metal complexes with d^4^ to d^7^ electron counts to reversibly switch between high‐spin (HS) and low‐spin (LS) electronic configurations in response to external stimuli. Most extensively studied in Fe(II) complexes possessing moderate ligand fields, the bistability of both HS and LS states in many SCO materials has fueled interest in a number of potential applications, including as sensors,^[^ ^27^ ^]^ molecular electronics and memory devices,^[^ ^28^ ^]^ actuators^[^ ^29^ ^]^ and for green applications such as barocalorics.^[^ ^30^ ^]^ Reversible HS ↔ LS switching is most commonly induced by temperature, pressure, or light (via Light Induced Excited Spin State Trapping, LIESST) however, other reported stimuli include magnetic^[^ ^31^ ^]^ and electric fields^[^ ^32^ ^]^ and chemically induced processes such as guest inclusion.^[^ ^33^ ^]^ Of most interest to the current discussion, SCO can also be mediated by ultrabright X‐rays.

Early studies into X‐ray‐induced SCO include work by Papanikolaou et al. in 2006 investigating the Prussian blue analogue (PBA) CsFe[Cr(CN)_6_] by synchrotron PXRD at the European Synchrotron Radiation Facility (ESRF) on beamline ID31.^[^ ^34^ ^]^ Magnetic susceptibility studies show CsFe[Cr(CN)_6_] displays a reversible HS ↔ LS transition on cooling/heating with a wide thermal hysteresis of ∼35 K. PXRD experiments on a pre‐cooled powder at 100 K indicated no significant damage from the synchrotron beam. However, on warming the sample to 245 K, where partial thermal reversion to the HS state has occurred (∼43:57% LS:HS ratio), prolonged X‐ray exposure at this warmer temperature resulted in an unexpected change. Significant peak shifts and broadening of the diffraction peaks associated with the HS phase were observed, with the indexed unit cell volume decreasing by 0.82(3)% after 6 min total exposure. This contraction on prolonged X‐ray irradiation was determined to result from an X‐ray induced HS → LS transformation that, interestingly, persisted on warming the powder to 265 K in the absence of further X‐rays. Heating above 265 K caused the sample to return to its ambient HS state; however, the X‐ray‐induced LS phase could be re‐accessed above this critical temperature on continuous illumination from the synchrotron beam, with the HS → LS kinetics becoming progressively slower the higher the temperature was raised. It is notable that this X‐ray‐induced transformation could be achieved at temperatures outside the thermal hysteresis loop, indicating the tantalizing potential to tune SCO with X‐rays near ambient conditions.

**Figure 3:**
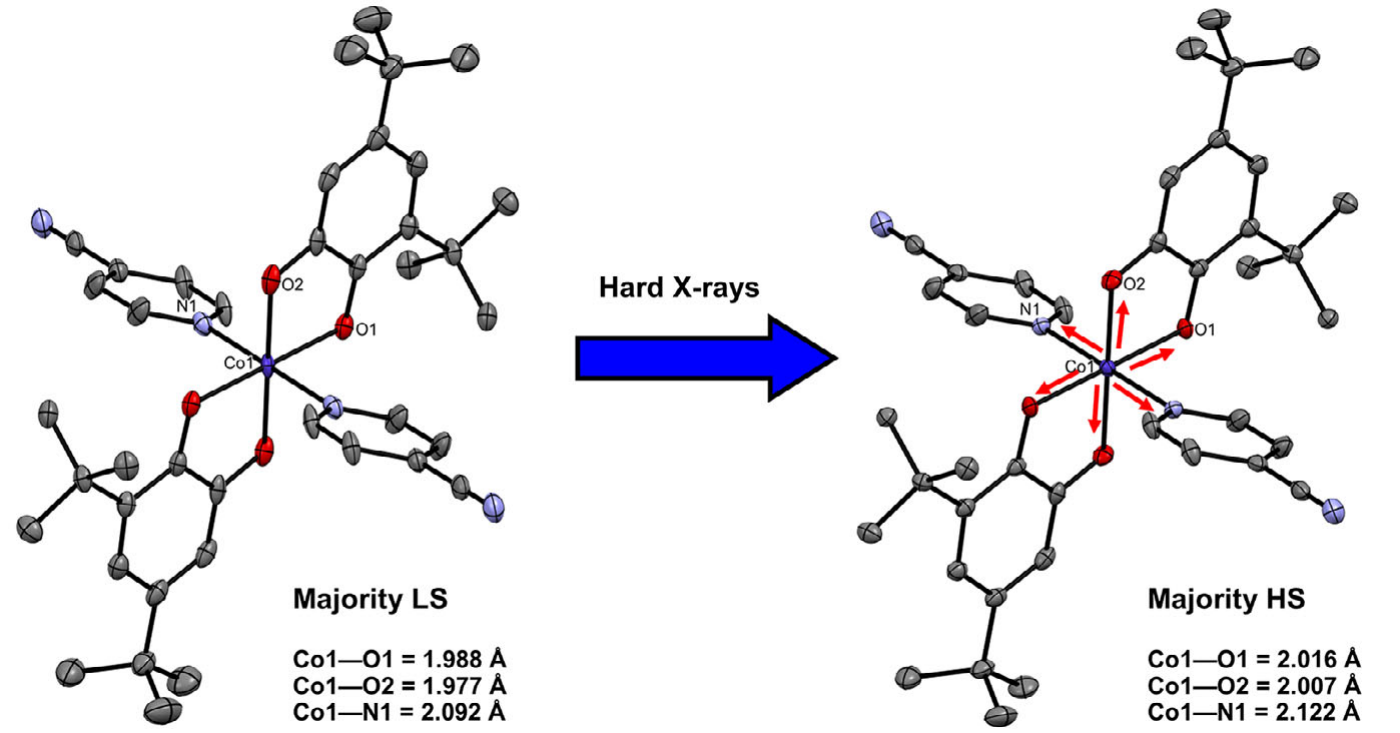
Hard (25 keV) X‐ray induced low spin (LS) → high spin (HS) state conversion in crystals of [Co(diox)_2_(4‐CN‐py)_2_] (diox = 3,5‐di‐t‐butylsemiquinonate, 4‐CN‐py = 4‐cyano‐pyridine) reported by Pinheiro, Raithby, and coworkers.The Co coordination sphere is observed to expand on prolonged exposure to the synchrotron X‐ray beam at 30 K. The majority LS single crystal X‐ray structure (left, CCDC refcode BIKZUT02) contains a majority LS population of 52% (48% HS) after a total experiment time of 30 minutes. The majority HS single‐crystal X‐ray structure (right, CCDC refcode BILBOQ02) contains a maximum HS population of 80% (20% LS) after a total experiment time of 301 minutes.^[^ ^35^ ^].^

More recently, in 2017, Pinheiro, Raithby, and coworkers reported X‐ray‐induced valence tautomerism in single crystals of the molecular complex [Co(diox)_2_(4‐CN‐py)_2_] (diox = 3,5‐di‐t‐butylsemiquinonate, 4‐CN‐py = 4‐cyano‐pyridine). Exposure to hard (25 keV) synchrotron X‐rays on beamline I19 at DLS was found to induce LS → HS switching in the Co center at 30 K, accompanied by intramolecular electron transfer between Co and the diox ligand (Figure 3).^[^ ^35^ ^]^ A maximum X‐ray‐induced population of 80% for the metastable ${\mathrm{Co}}_{HS}^{2+}$ redox isomer could be determined at 30 K, which was found to be rapidly reversible on warming the crystal back toward 100 K.

Returning to PBA materials, Boström et al. reported X‐ray‐induced SCO switching in microcrystalline powders of Fe[Pt(CN)_6_] during high‐pressure PXRD measurements on the P02.2 beamline at the PETRA III synchrotron in 2020.^[^ ^36^ ^]^ During initial variable‐pressure diffraction experiments, the HS state was found to be present up to 0.83 GPa, at which point both the HS and LS states were coexistent. Further pressure increases could deliver complete conversion to the high‐pressure LS state, which was found to be fully reversible on decompression. However, during a long exposure data collection, the influence of prolonged X‐ray irradiation on the SCO sample was serendipitously revealed. At the low pressure of 0.6 GPa, HS → LS conversion became evident after just 30 s of X‐ray exposure, reaching a maximum LS population of ∼85% after a total of 8 minutes of continuous irradiation. Following these measurements, a shorter control experiment was run on a pristine area of powder not previously exposed to the X‐ray beam, which was found to only contain the initial HS state.

Finally, Chernyshov et al. have studied the SCO complex [Fe(tame)_2_]^2+^ (tame = 1,1,1‐*tris*(aminomethyl)ethane) by SCXRD methods on the BM01 end station of the Swiss‐Norwegian Beamlines at the ESRF.^[^ ^37^ ^]^ Complete datasets were collected at 3 K intervals whilst the crystal was cooled from 260 K to 83 K. The authors attribute the unusual trends seen in the evolution of unit cell parameters to radiation damage from the synchrotron beam. Following an expected thermal contraction of the unit cell on initial cooling, at ca. 160 K an unusual anisotropic cell expansion was observed, to such an extent that the cell volume at 80 K was comparable to that determined at 240–250 K. The conclusion of X‐ray damage is further evidenced by significant increases in the anisotropic displacement parameters (ADPs) refined from the data recorded at lower temperatures, which correspond to the highest accumulated X‐ray dose levels, contrary to the expected decrease in ADPs at lower temperatures due to reduced thermal motion. In contrast to the studies discussed above, there is no significant evidence of X‐ray‐induced SCO switching in this molecular crystal system; however, on comparing their results with previously reported data for an analogous Cl complex,^[^ ^37b^ ^]^ the authors hypothesize that the themal HS → LS transition may be retarded as a result of the radiation effects.

### 2.3 Linkage Isomerism

**Figure 4:**
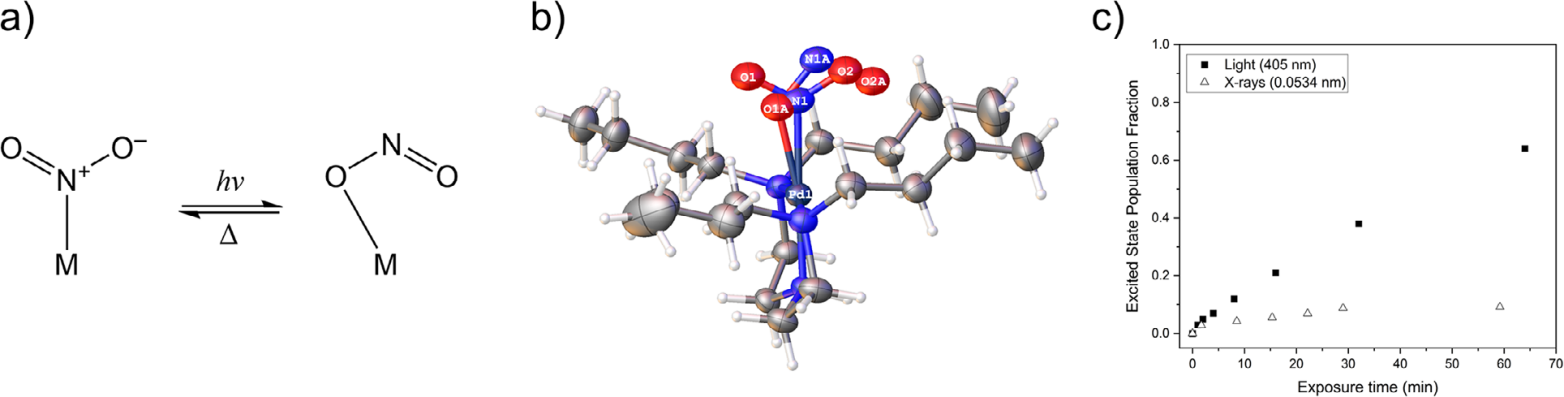
a) Schematic of nitro‐nitrito linkage isomer switching; **b)** single‐crystal X‐ray structure showing the asymmetric unit of a partially excited [Pd(Bu_4_dien)(NO_2_)][BPh_4_], showing the relative positions of ground‐state nitro‐(*η* 1‐NO_2_) and excited‐state *endo*‐nitrito‐(*η* 1‐ONO) linkage isomers; **c)** radiation‐induced linkage isomer switching in [Pd(Bu_4_dien)(NO_2_)][BPh_4_] as a function of exposure time for blue LED light (*λ* = 405 nm) and synchrotron X‐rays on beamline I19 at DLS (23 keV at 25% beam transmission).^[^ ^48^ ^].^

Linkage isomerism in transition metal complexes is a phenomenon by which an ambidentate ligand, containing more than one potential donor atom, can adopt multiple different coordination geometries. Examples of ligands known to form linkage isomer complexes include nitrosyl,^[^ ^38^ ^]^ nitrite,^[^ ^39^ ^]^ sulfur dioxide,^[^ ^40^ ^]^ dinitrogen^[^ ^41^ ^]^ and thiocyanate.^[^ ^42^ ^]^ In the crystalline state, switching between different isomer arrangements can be achieved on the application of external stimuli, most commonly light and temperature, with long‐lived metastable excited state isomers becoming cryotrapped when irradiation is performed at low temperatures (typically <150 K). There have been numerous examples of in situ SCXRD and PXRD studies of linkage isomer materials in the literature, and we refer the reader to the several comprehensive reviews already published.^[^ ^38^, ^39^, ^43^ ^]^ The seminal report of the first single‐crystal‐to‐single‐crystal photoinduced linkage isomer (PLI) transformation was published by Coppens et al. in the late 1990s for the archtypal photoswitch sodium nitroprusside.^[^ ^44^ ^]^ Complete, 100% PLI switching has been achieved in several crystal systems, most notably for nitro–nitrito PLI in group 10 transition metal nitrite complexes, such as Ni(dppe)(NO_2_)X (X = Cl, NO_2_)^[^ ^39^, ^45^ ^]^ and various Pd(II)‐triamines.^[^ ^46^ ^]^ PLI has been the benchmark on which the discipline of photocrystallography has been established in the last 30 years, experiments that are typically conducted at synchrotron and, more recently, XFEL facilities due to a requirement for small crystal volumes to maximise the penetration of the excitation light through the crystal bulk. PLI systems have also contributed to advances in the study of short‐lived photoinduced species by time‐resolved photocrystallographic methods.^[^ ^47^ ^]^ Despite the obvious challenge of prolonged exposure to both the intense X‐ray and light source beams, which is often repeated over multiple data collections, or even cyclically for pump‐probe time‐resolved studies, it is surprising that there are few reports of X‐ray damage or other induced processes in PLI systems to date. In 2022, Raithby et al. reported the development of a new pump‐multiprobe SCXRD methodology for the time‐resolved study of nitro‐nitrito PLI in the system [Pd(Bu_4_dien)(NO_2_)][BPh_4_] at millisecond timescales.^[^ ^48^ ^]^ Preliminary tests in the development of this methodology, performed on beamline I19 at DLS, included light and X‐ray damage testing and led to the discovery of an X‐ray‐induced nitro → nitrito transformation on prolonged X‐ray exposure using 25% of the available beam. A maximum conversion to 9.1% of the excited state *endo*‐nitrito‐(*η* 1‐ONO) isomer could be refined from the data after a total of 25 minutes of X‐ray exposure, which was identical to the excited state isomer geometry produced by visible light irradiation (Figure 4). Though 9.1% was considerably lower than the excited state population that could be accessed by visible light irradiation, this was still deemed potentially problematic for the pump‐multiprobe photocrystallography study at hand. Reducing the beam transmission to just 5% of the total available flux was found to minimize X‐ray excitation; however, a small baseline X‐ray conversion level is still observed in the final time‐resolved photocrystallography datasets. These results underline the importance of preliminary testing in the design of an in situ diffraction methodology to ensure that all potential variables are identified and controlled. They also reinforce the potential for unexpected X‐ray‐induced processes in functional chemical switches, particularly in those already predisposed to respond to other types of radiation.

### 2.4 Photochromism

**Figure 5:**
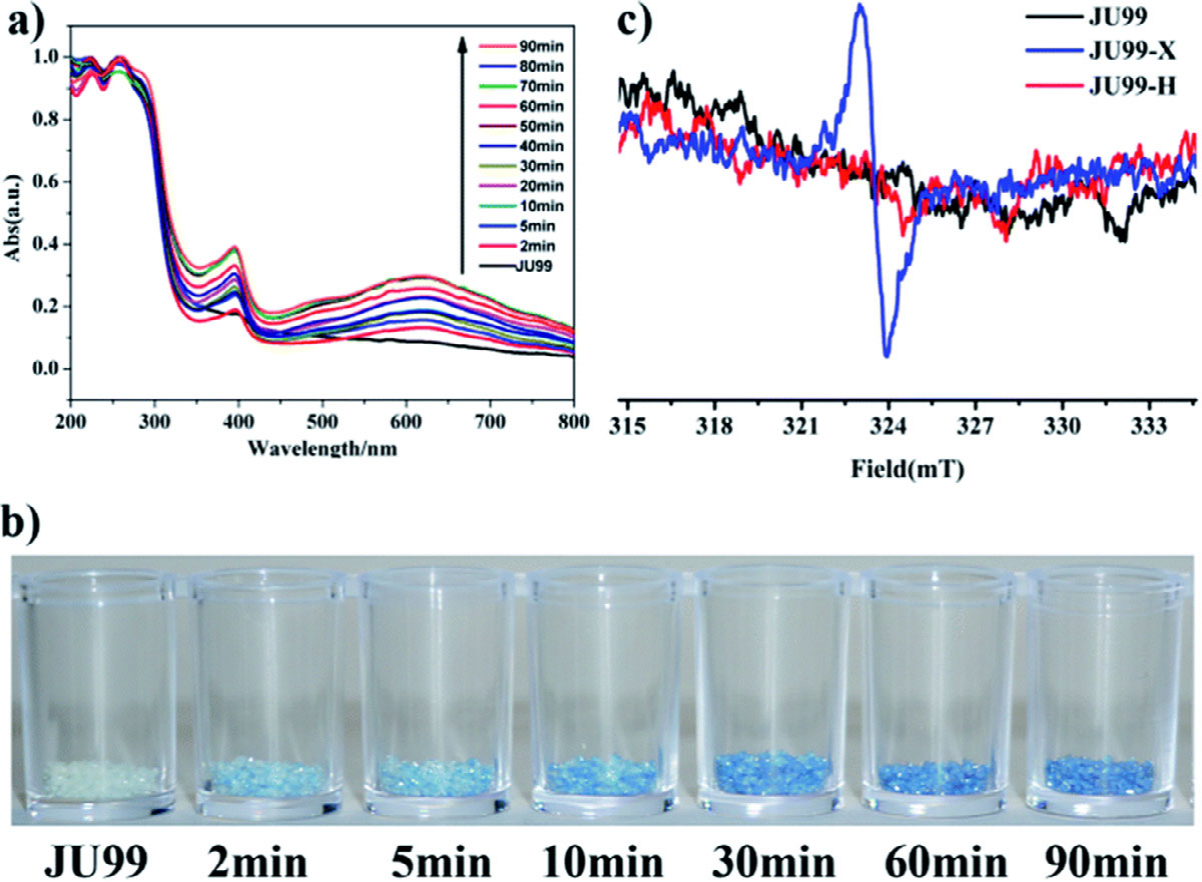
Data confirming X‐ray induced photochromism in the MOF JU99, a) evolution of the UV/vis spectrum of JU99 with X‐ray irradiation time, b) photograph of the photochromic changes following differing amounts of time exposed to Cu Ka X‐ray irradiation, c) EPR spectra confirming X‐ray induced changes (JU99‐X) and heat‐induced changes (JU99‐H) in samples dissolved in HCl solution. [Reprinted with permission from Wu et al., Chemical Science 2014, 5, 4237–4241. Copyright 2014 Royal Society of Chemistry.]^[^ ^51^ ^].^

Photochromic materials undergo a reversible transformation between two states that have different optical absorption properties and thus are characterized by distinct bulk color switching in response to excitation by a particular wavelength of light.^[^ ^49^ ^]^ This phenomenon has historically been studied primarily using wavelengths in the visible to UV region; however, in the last 15 years, an increasing number of articles are reporting X‐ray‐induced photochromism, with the materials under study showing great potential for applications in X‐ray sensing, including as scintillation counters or ionization gauges. In order to achieve this goal, there is much interest in designing materials capable of a rapid and quantifiable X‐ray response. Examples include the organometallic complex Zn(4,4′‐bipyridinium‐*N*‐propionate)Br_2_, studied by Wang et al. in 2012, which was unexpectedly observed to undergo an X‐ray‐induced color change during the course of an SCXRD collection using a standard laboratory Mo‐*K*_α_ X‐ray source (*λ* = 0.71073 Å) from yellow to blue. A similar transformation was also identified using Cu‐*K*_α_ radiation (*λ* = 1.54056 Å) for the collection of PXRD data.^[^ ^50^ ^]^ In other work by Wu et al., the layered MOF JU99 was designed to display photochromic properties due to the incorporation of a chromic methylviologen ligand within the framework pores.^[^ ^51^ ^]^ During successive PXRD measurements using a laboratory powder diffractometer equipped with a Cu‐*K*_α_ source, the MOF powder was observed to transform from colorless to a deep blue (Figure 5), with the blue phase stable in air for several months. The authors discovered that the colour change could be readily reversed upon heating the sample for several hours, leading to interest in the system as a bistable sensing device. Finally, a more recent report by Wang et al. in 2021 details X‐ray‐induced photochromism in a thulium‐based material, Tm(TPC)_2_(HCOO)(H_2_O) (TPC = 2,2′:6′,2′′‐terpyridine‐4′‐carboxylate). The rare‐earth complex was found to display a strong photochromic response to both UV light and X‐ray exposure, leading the authors to postulate its use for future X‐ray dosimetry devices.^[^ ^52^ ^]^

### 2.5 Other Unit Cell Changes and Phase Transitions

Significant changes in the crystallographic unit cell parameters in response to X‐ray irradiation have not only been reported in parallel with SCO switching, as in the previous example by Chernyshov et al., but also in a range of other materials, including organometallic catalysts,^[^ ^53^ ^]^ coordination polymers,^[^ ^54^ ^]^ and thermoplastic precursors.^[^ ^55^ ^]^ A handful of recent articles are discussed in detail in this section.

**Figure 6:**
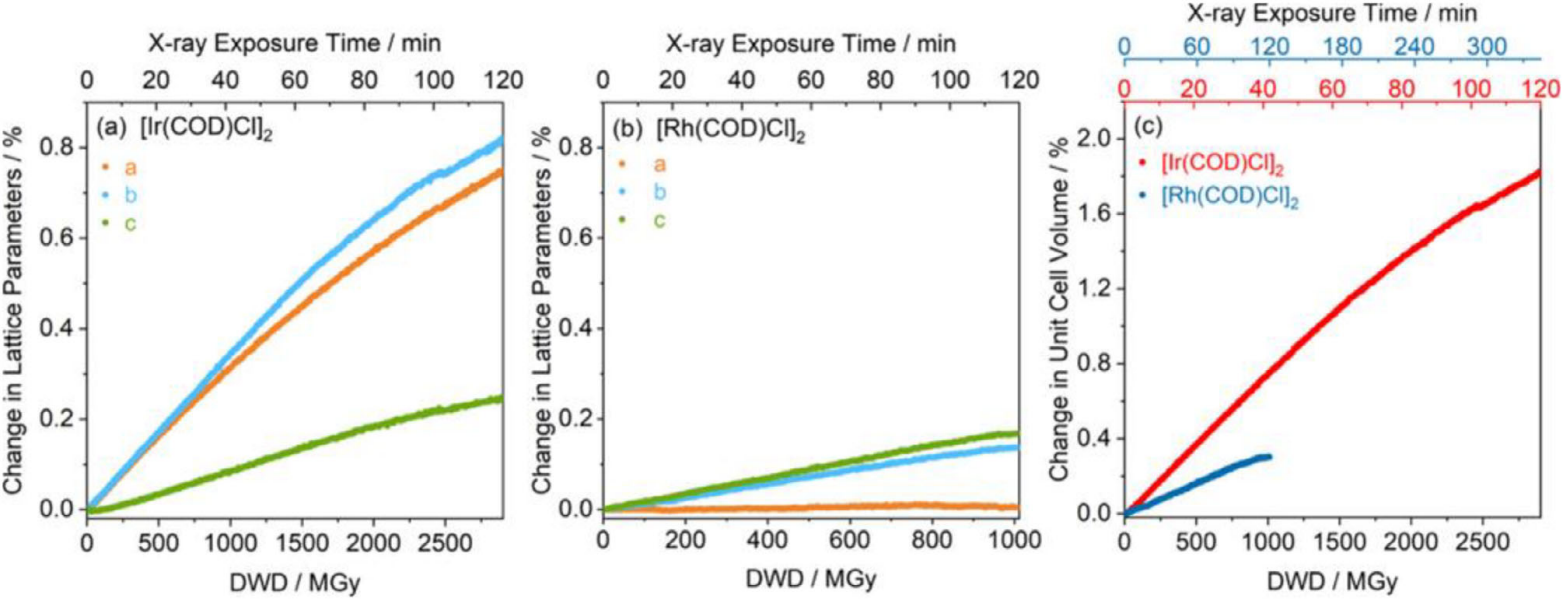
Percentage change of catalyst unit cell parameters as a function of accumulated X‐ray dose; a) lattice parameters for [Ir(COD)Cl]_2_ and b) lattice parameters for [Rh(COD)Cl]_2_, and c) unit cell volume for both catalysts. The maximum change in refined unit cell volume for each complex was determined as ΔV = +1.9% for [Ir(COD)Cl]_2_ (2903 MGy dose) and ΔV = +0.3% for [Rh(COD)Cl]_2_ (1012 MGy dose). [Reprinted with permission from N. K. Fernando et al*., The Journal of Physical Chemistry A* 2021, 125, 7473–7488. Copyright 2021 American Chemical Society.]^[^ ^53a^ ^].^

In 2021, Fernando et al. reported the influence of X‐ray irradiation on the structural and electronic properties of the industrially relevant catalysts [Ir(COD)Cl]_2_ and [Rh(COD)Cl]_2_ (COD = cyclooctadiene) by X‐ray photoelectron spectroscopy (XPS) and synchrotron PXRD. In this study, the authors utilized the program *RADDOSE‐3D* to quantify the changes seen as a function of absorbed dose.^[^ ^53a^ ^]^ On beamline I11 at DLS, 500 PXRD patterns were collected on a sample of each of the catalysts. For [Ir(COD)Cl]_2_, a rapid decrease in the diffraction peak intensities was observed with increasing X‐ray dose, accompanied by significant peak broadening and a general shift of all peak positions toward lower 2θ, indicating an expansion of the unit cell with prolonged exposure to the synchrotron beam. Conversely, the changes seen in the patterns collected on the Rh analogue are considerably less dramatic, although the general behavior tends toward a similar trend to that of the Ir complex. Le Bail refinements on the high‐resolution PXRD patterns first provided more quantitative insight, showing that in [Ir(COD)Cl]_2_ the unit cell expansion was partially anisotropic, with the *a* and *b* axes increasing by ca. 0.8% and the *c* axis expanding only by 0.2% after a total X‐ray dose of 2903 MGy. By contrast, refinements on the patterns collected for [Rh(COD)Cl]_2_ confirmed the interpretation that this complex is considerably less susceptible to the X‐ray beam, with the *b* and *c* axes increasing by < 0.2% and very little change in the *a* parameter for a total received dose of 1012 MGy. The quantitative trends in the unit cell parameters as a function of dose are summarized in Figure 6 below, which further confirms the significantly different responses of the two catalysts. To enable atomic‐scale analysis of the X‐ray induced structure changes, structure solution via Rietveld analysis was performed on each set of data. The greater response to X‐ray irradiation by the [Ir(COD)Cl]_2_ complex is manifested in a significant expansion of the Cl‐Ir‐Cl angles for the two bridging μ^2^‐Cl ligands, by 6.4% and 8.3%, while the Cl…Cl distance across the dimer increases by 5.2%. By comparison, analysis of the equivalent Cl‐M‐Cl angles and Cl…Cl separation in the Rh catalyst reveals near negligible changes. While PXRD analysis is capable of showing the crystal structure changes in response to irradiation as a spatial average across the bulk sample, this study also marks the first reported use of RADDOSE‐3D for XPS measurements. Valence band and core‐level XPS spectra were used to investigate how the X‐ray‐induced structure changes are reflected in the electronic structure, with the observed spectra displaying features consistent with partial X‐ray photoreduction and changes in the chemical environment of the metals for both catalysts. This comprehensive study makes considerable progress toward understanding the global impact of X‐ray damage at the crystal, chemical, and electronic structure scales and concurrently reminds us of the need to employ multiple complementary analysis techniques to deliver a thorough interpretation of complicated phenomena.

In a follow‐up study, Fernando et al. conducted further experiments on [Rh(COD)Cl]_2_ in which they varied the X‐ray photon energy from 18 keV in the prior study to 8, 15, and 25 keV. This series of experiments aimed to investigate the impact of the experiment strategy and key controllable variables on the X‐ray‐induced structure changes and sought to identify the optimal settings for the greatest diffracted signal per dose.^[^ ^53b^ ^]^ Systematic studies into the energy dependence of X‐ray damage in the system were hampered by the inability to deliver consistent dose and dose rates across the different X‐ray energies, with the dose absorbed by the sample in equivalent experiments at 8 keV significantly higher than at 15 and 25 keV due to the beamline setup. Despite this, the results highlighted the importance of developing dose‐related experiment protocols for the study of small molecule systems in high‐energy X‐ray beams. This conclusion will only become more important for synchrotron experimenters across the next decade, as facilities continue to upgrade their instrument capabilities.

The unusual observation of unit cell contraction as a result of X‐ray damage was investigated by Goodwin and coworkers in 2021, on the flexible coordination polymer cadmium cyanide (Cd(CN)_2_). The authors named this new effect negative X‐ray expansion (NXE) to align with the analogous process of negative thermal expansion (NTE) and defined the X‐ray expansion coefficient, σ, to quantify this X‐ray‐induced change.^[^ ^54^ ^]^ NXE in Cd(CN)_2_ was serendipitously discovered during the authors attempt to reconcile conflicting prior neutron and X‐ray investigations of the low temperature phase transition in this material. In synchrotron PXRD studies on beamline I11 at DLS, a series of repeat powder patterns were collected on a microcrystalline sample held at 200 K. These isothermal repeat measurements revealed a gradual cell contraction on prolonged X‐ray exposure, indicating an X‐ray induced transformation had occurred. The authors confirmed the changes were not due to local heating (which could alternatively suggest NTE behavior) by collecting reference variable temperature PXRD data. A second X‐ray‐induced effect was identified on cooling a fresh sample to 100 K, which expectedly induced the low‐temperature symmetry‐breaking phase transition from the parent cubic $Pn\bar{3}m$ phase. Prolonged X‐ray exposure at 100 K produced a different response to that seen at 200 K, with the X‐rays unexpectedly initiating a reverse transformation from the (disputed) low‐temperature phase back to $Pn\bar{3}m$. The authors note that, as for many materials displaying complex phase behavior in response to multiple stimuli,^[^ ^56^ ^]^ the specific combination and, particularly, the order in which different stimuli are applied is important, as different procedures can provide access to different parts of the phase space, resulting in distinct product phase outcomes. The discovery of this interesting new X‐ray induced phenomenon was followed up shortly after by Boström et al. in 2022. They identified a small, yet significant, unit cell contraction in the multi‐stimuli responsive PBA CsMnCo(CN)_6_ as a function of X‐ray dose, using synchrotron PXRD data collected on beamline I11 at DLS.^[^ ^57^ ^]^ These results again hint at the exciting potential to tune material properties using X‐rays as a pump rather than a probe source, opening interesting avenues for future exploration.

On a similar theme of negative expansion behaviors, in 2022 the organic thermoplastic precursor pyromellitic dianhydride (PMDA) was studied by Porȩba et al. on beamlines ID15B and ID22 at the ESRF.^[^ ^55^ ^]^ This material is known to be sensitive to both γ‐ray and X‐ray irradiation, with the latter previously reported in PMDA thin films on irradiation at 15 keV.^[^ ^58^ ^]^ In their study, Porȩba et al. first identified a reversible thermal phase transition at ∼210 °C in polycrystalline PMDA by differential scanning calorimetry (DSC), confirmed to be a symmetry‐breaking transformation by complementary Raman collections. They then conducted in situ SCXRD studies at variable temperature to explore the structural basis for the phase change. Unusually, the crystal was first loaded into a diamond anvil cell (DAC) in some iterations of the temperature study in an attempt to recreate the conditions for DSC. A limited region of NTE was identified at ca. 141 °C with traditional positive thermal expansion observed at temperatures below and above this region. Single‐crystal integrity was then lost above 219 °C, close to the expected phase transition point from DSC. The study was continued using PXRD, with Le Bail fits for the patterns recorded above the transition temperature revealing a tetragonal to monoclinic phase change. X‐ray damage was identified during repeat isothermal measurements and is found to accelerate as the temperature is raised. In this case, the occurrence of radiation damage proved to be a limiting factor for the investigation, preventing collection of PXRD data to the high‐resolution required for structure determination of the new, high temperature monoclinic phase.

**Figure 7:**
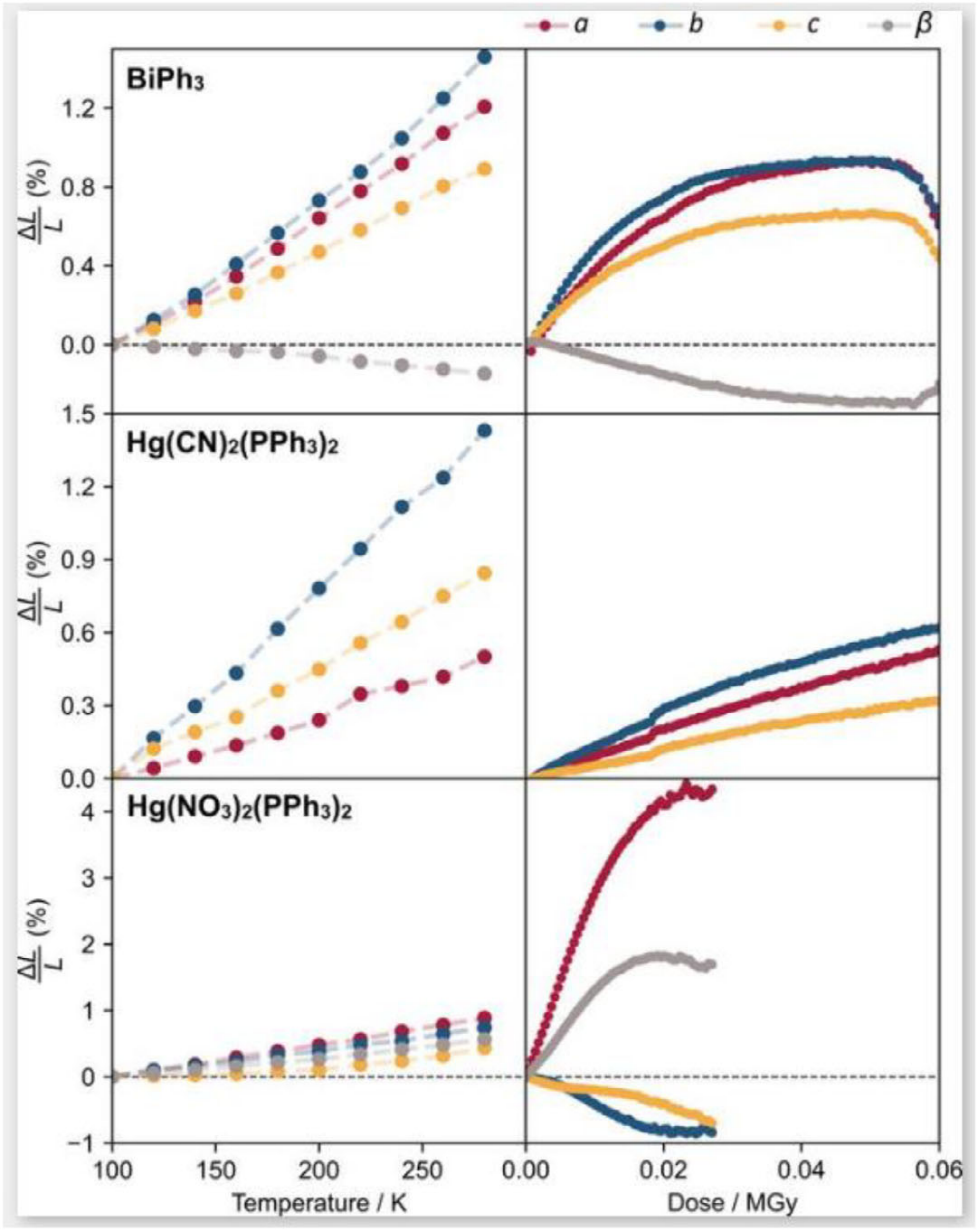
Change in unit cell parameters for the radiation‐sensitive metal phosphines BiPh_3_, Hg(CN)_2_(PPh_3_)_2_ and Hg(NO_3_)_2_(PPh_3_)_2_ as a function of temperature and accumulated X‐ray dose. The unit cell parameters are plotted as a percentage of the relative difference to their values at 100 K at zero dose. [Reprinted with permission from McMonagle et al*., Acta Crystallographica Section B* 2024, 80, 13–18. Copyright 2024 International Union of Crystallography]^[^ ^59a^ ^].^

Finally, while many of the studies reviewed in this article have used dose calculations to quantify X‐ray absorption by a susceptible sample, work by McMonagle et al. in 2024 shows how a material's physical response to radiation can in fact be used for indirect quantification when experiments are performed in a systematic and controlled way. In this study, the authors build on the work of Goodwin et al.^[^ ^54^ ^]^ to define a radiation‐induced strain tensor to quantify the level of X‐ray damage in a crystal. The concept is benchmarked against three radiation sensitive metal‐phosphine crystals, with SCXRD experiments performed on the BM01 end station of the Swiss‐Norwegian beamlines at the ESRF. The total X‐ray dose absorbed by the crystals during each data collection was calculated using *RADDOSE‐3D*, providing a comparative measure for the authors to benchmark their strain tensor method against. A series of repeat, isothermal SCXRD collections were performed on each sample to accumulate the X‐ray dose, with series repeated at regular temperature intervals between 100 and 300 K. Figure 7 summarizes the unit cell dimensions recorded in this study as a function of temperature and total absorbed dose. These data show that there are considerably different responses across all materials with respect to temperature and accumulated dose and, while BiPh_3_ and Hg(CN)_2_(PPh_3_)_2_ generally show an expected expansion in all lattice parameters on both heating and prolonged X‐ray exposure, the nitrate complex Hg(NO_3_)_2_(PPh_3_)_2_ shows a considerably more anisotropic response to radiation, with a large expansion in the *a* direction but compression along *b* and *c*. To adequately quantify the anisotropic lattice response to X‐ray exposure, the authors develop a second‐rank strain tensor for the radiation‐induced change, in analogy with those developed by other researchers to quantify thermal‐or pressure‐induced lattice expansion.^[^ ^59^ ^]^ The authors note the limitations of their approach in requiring high‐quality data that is most likely only obtained for the situation of mild X‐ray damage or under controlled conditions where the experimenter is intentionally targeting the quantification of a known X‐ray‐induced phenomenon. That said, this approach offers an alternative route to quantify radiation damage in situations where performing dose calculations may be challenging. This includes in situ studies, where the use of specialist environmental cells or the need for multiple stimuli or probe radiation types may prevent determination of the sample and X‐ray beam properties required to parameterize dose calculator tools. As these types of in situ experiments are likely to be some of the worst affected by induced damage processes, both from the X‐ray probe and from other sources (e.g., excitation stimuli such as lasers, applied fields, or chemical triggers), this approach is certainly noteworthy.

## 3 Innovative Methodological Approaches To Address Radiation Damage

In accepting the mounting evidence that X‐ray radiation can no longer be considered an innocent probe for the study of chemical crystalline solids, small molecule crystallographers need to be vigilant and innovative in their approach to identify X‐ray‐induced processes and address their impact on the solid‐state properties under study. When radiation damage compromises the outputs of an investigation, routes to minimize or eliminate the problem must be considered. Common approaches to mitigate X‐ray damage that can be achieved easily using a standard diffraction set‐up include: i) reducing the temperature, that is, cryocooling the sample to retard the movement of the photoelectrons and free radicals that propagate damage; ii) attenuating the X‐ray beam; and iii) minimizing the X‐ray exposure time, both to reduce the total X‐ray dose accumulated. For protein crystals, cryoprotectant liquids are also commonly used that can help to minimize additional radiation damage by preventing the formation of ice (and thereby reducing a potential source of hydroxyl radicals) during rapid cryocooling. Although this approach is unnecessary for many chemical samples, cryoprotectants could be considered for crystals containing a high percentage of solvent (e.g., porous materials) and those that are hygroscopic or otherwise moisture sensitive. Furthermore, on instruments offering the option to control X‐ray beam parameters, matching the beam size to the crystal dimensions can also be beneficial. This approach helps to maximize the signal‐to‐background ratio, improving the data quality while reducing unnecessary exposure, thereby helping to mitigate radiation damage.^[^ ^60^ ^]^ Where these resolutions are either insufficient to protect the crystal or are not compatible with the experiment to be run, for example, during in situ or time‐resolved experiments where particular experimental factors are imposed, then new methodological solutions must be designed.

This section explores the innovative methodological approaches that have developed to address radiation‐induced effects in chemical crystalline materials, illustrating each discussion with a few notable examples. It is not intended to be an exhaustive review of all of the reports utilizing each method but aims to provide a concise summary of the options available to chemical crystallography researchers to minimize the impact of X‐ray damage in their data.

### 3.1 Multi‐Sampling Methods

One rational approach to reduce X‐ray damage is to spread out the total dose required for a data collection over multiple sampling areas. Such a method is already routinely applied in synchrotron PXRD studies, where it is common to translate the sample capillary perpendicular to the beam between repeat data collections, even when there is no specific concern over X‐ray‐induced effects. Translation is particularly employed for in situ experiments, such as variable temperature measurements to study phase transition behavior,^[^ ^61^ ^]^ where it would be impractical to change capillaries between every separate collection in the temperature series. This methodology has been utilized in many of the PXRD studies discussed in Section 2, including during the studies of NXE in Cd(CN)_2_ by Coates et al.^[^ ^54^ ^]^ and the multi‐responsive PBA CsMnCo(CN)_6_ by Boström et al.^[^ ^57^ ^]^ For this approach to be effective, care must be taken to understand the width of the radiation‐affected region along the capillary, considering both the X‐ray beam size in use and also the wider propagation of any damage effects along the capillary. For example, during their study of the radiation‐induced effects in pyromellitic dianhydride (PMDA), Porȩba et al. observed that prolonged X‐ray exposure when the sample was placed inside a sealed capillary near room temperature for ca. 3 hours led to X‐ray‐induced recrystallization, producing large single crystals of PMDA from the starting microcrystalline powder.^[^ ^55^ ^]^ Thus, the authors also utilized sample translation when investigating the sample's thermal properties in capillary mode to decouple their results from any X‐ray‐induced effects. For problematic materials, special loading techniques could be considered to minimize contamination from the X‐ray‐damaged portion of the sample, for example, by filling alternate sections of the capillary with powder sample and a spacer material (e.g., cotton or glass wool).^[^ ^62^ ^]^

By analogy with this multi‐sampling approach for capillary PXRD, the total X‐ray dose for an SCXRD experiment can be similarly spread out in space by rastering over multiple positions on one large single crystal as it is translated across the X‐ray beam. This is a similar approach to that used in spatially resolved spectroscopic studies, such as Raman mapping.^[^ ^63^ ^]^ Such analyses require the use of small beam sizes, usually micro‐ or even nanofocus X‐ray beams, and thus are typically confined to synchrotrons at beamlines that are optimized for high spatial resolution studies. Examples of the required methodology include a study by Fischetti and coworkers in 2010, who used a selection of microfocus beam sizes available on beamline 23ID‐B at the Advanced Photon Source (APS) to perform spatially resolved measurements across a large single crystal of lysozyme, mapping the spatial extent of X‐ray damage from the focused synchrotron beam.^[^ ^64^ ^]^ From their results, they argued that the use of smaller X‐ray beams can in itself be a route to reduce X‐ray damage in protein crystals, with the rate of damage per normalized X‐ray dose decreasing in correlation with the beam size. Another study on a similar topic by Shepherd et al., conducted on beamline PROXIMA 2A at Synchrotron SOLEIL in 2016, showcased the use of helical data collection scans along long, thin needle crystals as a route to mitigate radiation damage effects.^[^ ^65^ ^]^ By comparing the data collected via standard versus helical scan methods, the authors prove conclusively that helical scans are successful in reducing radiation damage in the two proteins studied: human transthyretin and human matrix metalloproteinase 12, producing electron density maps of superior quality as a direct result of spreading the total X‐ray dose out along the length of the needle. Although this solution for radiation damage has primarily been used to study protein samples to‐date, likely due to their stronger perceived susceptibility to X‐ray damage and the greater awareness of radiation‐induced effects among the MX community, in principle there is no reason why it cannot be adapted for the study of chemical crystals. Indeed, in many cases, large single crystals of molecular materials are more readily accessed than for proteins, and so this could be a viable route to mitigate X‐ray damage in many chemical crystals at accelerator facilities. Alongside the discussed enhancements in X‐ray brilliance, improvements in the beamline optics at many upgraded (or upgrading) synchrotron sources are also resulting in the increased global provision of micro‐and nanofocus instruments, which could make these spatially resolved approaches even more viable in the near future.

### 3.2 Multi‐Crystal Methods

Rather than spreading the total X‐ray dose required for the experiment across multiple areas of the same sample, the alternative is to instead spread the dose over many different samples. As outlined in the introduction, multi‐crystal approaches have been developing at pace over the last 15 years as a direct result of the unsustainable levels of radiation damage inflicted on single crystals by ultrabright accelerator sources. In particular, “diffract‐before‐destroy” SX experiments were first developed for protein structure determination at XFELs. These SFX experiments make use of the femtosecond time structure of the FEL to collect room temperature structure data quickly before a susceptible protein crystal can degrade via one of several common mechanisms, for example, due to dehydration on exposure to air or from X‐ray damage.^[^ ^7^ ^]^ In these experiments, a stream of many thousands of individual crystals must be delivered into the X‐ray beam such that, ideally, only a single diffraction image is collected on any one crystal. Complete data for structure determination is then accumulated by combining the best diffraction images collected from the many crystals that are exposed over the course of the SX experiment. Sample delivery is typically achieved by one of two key methods. Liquid or viscous jet delivery methods involve the suspension of the target crystals into a suitable carrier fluid before the crystal‐containing suspension is then injected into the beam. This option presents some challenges, including the need for crystals to be delivered at a consistent rate for the duration of the experiment and the propensity for clogging in the jet nozzle.^[^ ^66^ ^]^ Fixed target methods have developed as an alternative, particularly for serial synchrotron crystallography (SSX) approaches. Here, thousands of microcrystals are applied to a substrate, typically a specifically designed grid array, which is then rastered in a fast sequence across the X‐ray beam.^[^ ^67^ ^]^ SX collections for protein crystals at both synchrotrons and XFELs are now approaching routine operation and are increasingly being combined with in situ environments to enable real‐time determination of the structural dynamics in stimuli‐induced processes such as photolysis^[^ ^68^ ^]^ and *cis‐trans* photoisomerism.^[^ ^69^ ^]^ By contrast, SX studies on chemical crystalline materials are comparatively in their infancy, with only a limited number of studies in the literature to date, all performed since 2022. Despite this, interest in this nascent area of small molecule serial crystallography (smSX) is growing at both synchrotrons and XFELs, and the below sections summarize the progress being made at each source type, with particular focus on multi‐crystal methods to address X‐ray damage. A summary of the experiments discussed herein is provided in Table 1.

**Table 1:** Summary of the smSX experiments discussed in this section.

Publication Year	Corresponding Author(s)	Source Type	Sample Delivery	Material(s) Studied	Special Details [data collection or processing]	Citation [this text]
2022	J. N. Hohman	XFEL	Liquid / viscous jet	Silver benzenechalcogenolate hybrids	Sparse indexing approach based on PXRD methods	[71]
2023	J. N. Hohman	XFEL	Liquid / viscous jet	Silver n‐alkanethiolates	Sparse indexing approach based on PXRD methods	[72]
2023	B. B. Iversen	XFEL	Liquid / viscous jet	K_4_[Pt_2_(P_2_O_5_H_2_)_4_]·2H_2_O	Sparse indexing approach based on known unit cell parameters	[73]
2023	K. Yonekura	XFEL	Fixed target	Organic dye (rhodamine‐6 G)	Sparse indexing approach based on known unit cell parameters from prior 3DED experiments	[74]
2024	H. Ihee	XFEL	Fixed target	Metal organic framework (PCN‐224(Fe)‐CO)	14.5 keV X‐rays compress sparse diffraction pattern, allowing ab initio cell indexing from single images	[75]
2024	E. Collet	Synchrotron	Liquid / viscous jet	Prussian blue analogue (Rb_0.94_Mn_0.94_Co_0.06_[Fe(CN)_6_]_0.98_·0.2H_2_O)	Sparse indexing approach based on PXRD methods	[76]
2024	W. Shepard	Synchrotron	Fixed target	Metal organic frameworks (MIL‐100(Fe) and ZIF‐8)	Small wedge data collection approach achieved by small angle rotation of fixed target	[77]
2024	L. E. Hatcher, M. R Warren	Synchrotron	Fixed target	Sodium nitroprusside dihydrate (Na_2_[Fe(CN)_5_(NO)].2H_2_O)	Small wedge data collection approach achieved by small angle rotation of fixed target	[78]

#### 3.2.1 Multi‐Crystal Approaches at XFEL Sources

Despite the fact that there is considerably more research into chemical crystalline materials at synchrotron facilities, as for the original protein SX developments in the early 2010s, the majority of smSX method development to date has been conducted at XFELs. There is already a comprehensive review summarizing the development of smSFX methodologies to 2024, which describes in detail the breakthroughs made for both static structure determinations and time‐resolved approaches to study structural dynamics.^[^ ^70^ ^]^ This article also details the various data processing strategies employed in the smSFX studies discussed, including the software and algorithms employed. As such, we here discuss only a handful of examples in detail, focusing particularly on those using smSFX to study X‐ray‐sensitive materials and those whose study is hampered by other approaches.

**Figure 8:**
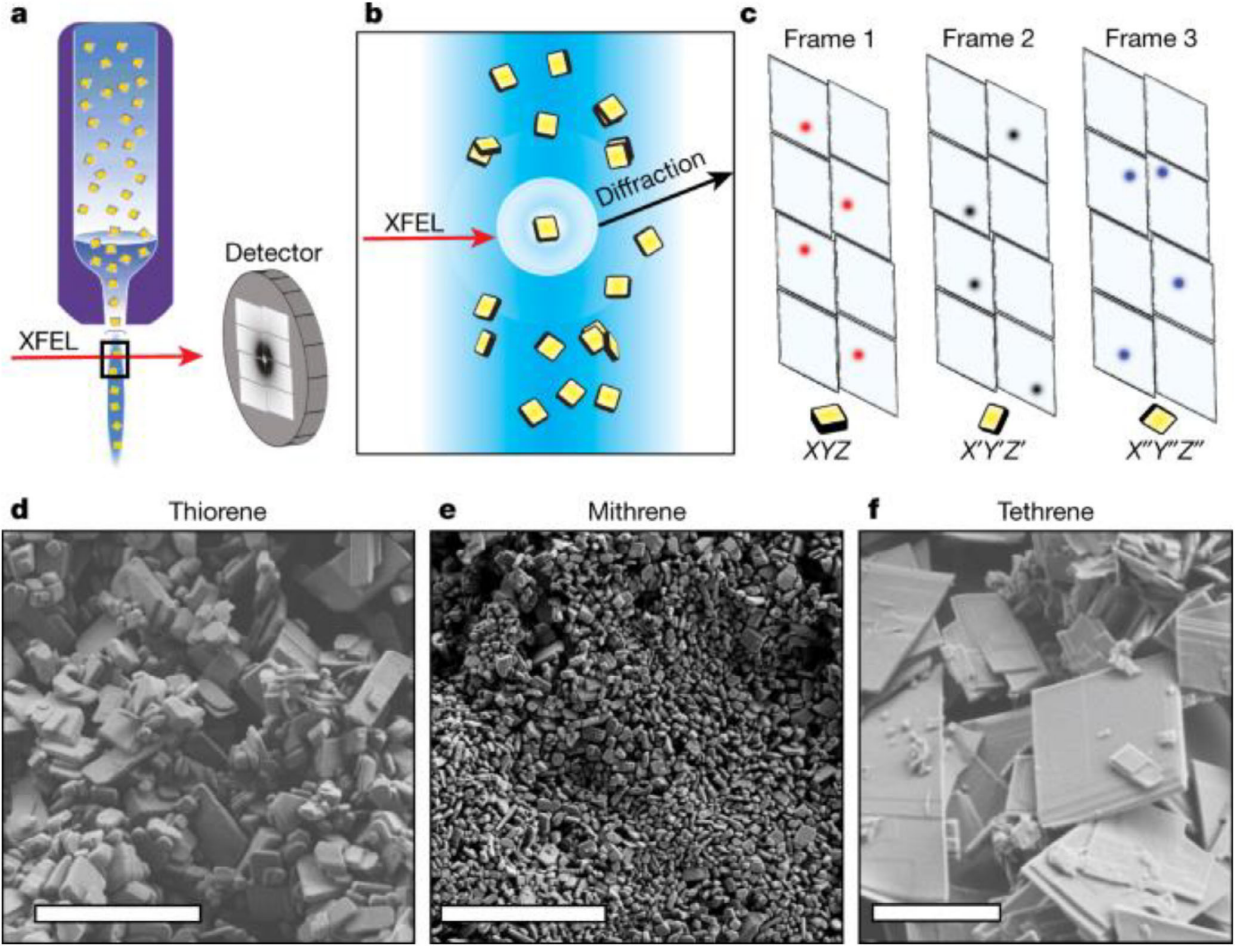
a)‐c) Schematics providing an overview of the first smSFX experiment performed by Hohman and coworkers to study microcrystals of three X‐ray‐sensitive silver benzenechalcogenolate hybrids; **d)‐e)** scanning electron micrographs of each microcrystal batch used for the experiment, scale bar = 5 µm. [Reprinted with permission from Schriber et al*., Nature* 2022, 601, 360–365, under a Creative Commons Attribution 4.0 International License:  http://creativecommons.org/licenses/by/4.0/. Copyright 2022 Springer Nature.]^[^ ^71^ ^].^

One of the key factors preventing the direct translation of protein SX methodologies for small molecules is the fact that chemical crystals in general display significantly smaller unit cell dimensions compared to proteins. This leads to sparse diffraction patterns,^[^ ^70^ ^]^ which in turn limits the number of Bragg diffraction peaks that can be recorded on the single image collected from each crystal. There are typically too few Bragg peaks per image to perform reliable indexing procedures, preventing determination of that crystal's unit cell parameters and thus its orientation with respect to the diffractometer axes. Crucially, this makes it impossible to merge together the data from all of the individual crystals, preventing final crystal structure determination by smSX. Addressing this issue has obviously been a particular focus for the pioneering smSFX studies reported in the last 3 years. The first reported smSFX study was published in January 2022 by Hohman and coworkers and applied the technique to three X‐ray‐sensitive silver benzenechalcogenolate hybrid materials: mithrene (AgSeC_6_H_6_), thiorene (AgSC_6_H_6_), and tethrene (AgTeC_6_H_6_).^[^ ^71^ ^]^ These materials had previously presented significant challenges for structure determination using either X‐ray or electron diffraction, with only the structure of mithrene previously recorded by SCXRD, despite attempts to study the other two systems by powder and single‐crystal approaches. In this seminal work, the authors were able to determine the 3D crystal structures of thiorene and tethrene for the first time, using an smSFX approach. Microcrystals of each of the three target materials were delivered into the XFEL beam on the Coherent X‐ray Imaging endstation at the Linac Coherent Light Source (LCLS) using a liquid jetting approach (Figure 8), allowing the collection of SFX datasets. To overcome the difficulties in indexing the individual images, Hohman et al. took inspiration from PXRD to devise a data processing solution in which they first summed the collected diffraction images to generate powder diffractograms from the complete SX dataset obtained across all crystals. They were then able to index these powder patterns to determine the unit cell parameters using standard PXRD indexing algorithms. These cell parameters were then imported back into the SFX data processing, enabling the determination of each crystal structure. Among the important results of this study, the smSFX crystal structure of thiorene revealed an unexpected difference in the Ag…Ag bonding network compared to the known structure of the Se analogue mithrene, which was used to rationalise thiorene's differing optoelectronic behavior. As such, the authors proved the benefits of smSFX to overcome the radiation sensitivity issues hindering the study of these important functional hybrids, delivering 3D structural insight to explain key structure‐property correlations not accessible via traditional approaches. This illustrates the exciting potential of smSFX to deliver atomic‐scale insight that can subsequently inform the design of new and improved functional materials, targeting specific applications.

**Figure 9:**
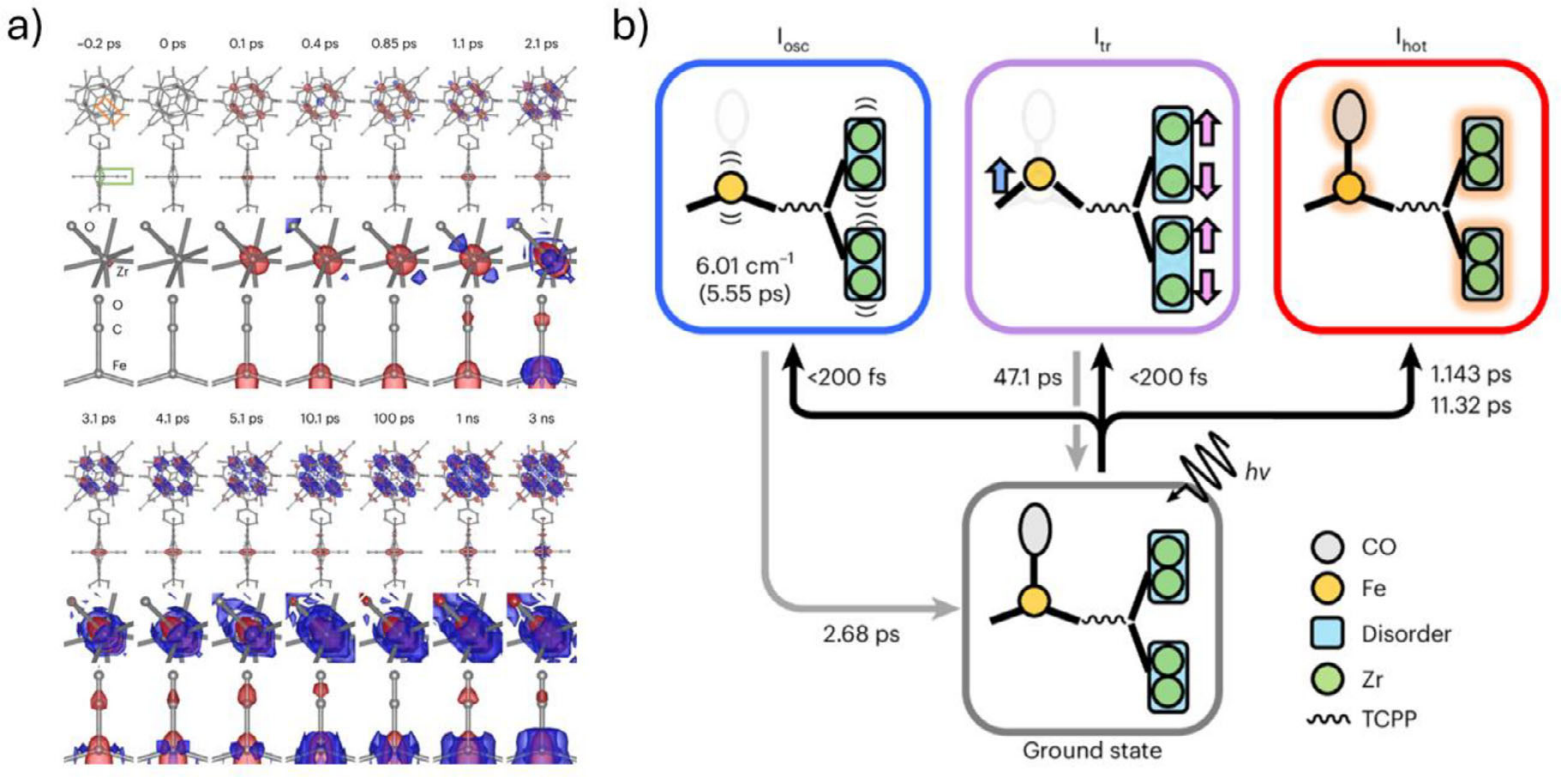
Results of the dynamic smSX study performed by Ihee and coworkers on the photoactive MOF PCN–224(Fe)–CO, a) difference electron density maps of the Fe porphyrin and Zr_6_ node (first and fourth row), one of the Zr atoms in the Zr_6_ node (second and fifth row), and the Fe porphyrin region (third and sixth row). Blue and red regions denote a positive and negative electron difference, respectively; b) an illustrative diagram to show the two anisotropic movements (the coherent oscillation and transient structure denoted by I_osc_ and I_tr_, respectively) along with the vibrationally hot structure (I_hot_) and the associated timescales for the emergence and decay of the structures in response to photoirradiation. [Reprinted with permission from Kang et al*., Nature Chemistry 2024, 16, 693–699*, under a Creative Commons Attribution 4.0 International License:  http://creativecommons.org/licenses/by/4.0/. Copyright 2024 Springer Nature.]^[^ ^75^ ^].^

Hohman et al. have since applied their smSFX approach to study the structures of other X‐ray‐sensitive Ag‐based hybrid crystals.^[^ ^72^ ^]^ In parallel, other research groups have been developing alternative sparse diffraction pattern indexing solutions for smSFX. In January 2023, Iversen et al. showed how the difficulty in indexing can be alleviated if the required unit cell parameters are already known from previous study.^[^ ^73^ ^]^ While Yonekura and coworkers took this route a step further in December 2023 by proving the benefits of multi‐technique approaches. Here, the authors first used 3D electron diffraction methods to obtain starting unit cell parameters for the organic dye rhodamine‐6 G, which they could then apply to an smSFX dataset to determine the molecular crystal structure.^[^ ^74^ ^]^ Most recently in 2024, Kang, Ihee, et al. have shown that the indexing challenge can be circumvented entirely for some chemical crystalline materials that display moderate unit cell dimensions, for example, extended materials such as metal‐organic frameworks, providing the experiment is performed at a facility with access to harder X‐ray energies.^[^ ^75^ ^]^ In this work, the authors performed the first dynamic smSFX study to investigate microcrystals of the photoresponsive iron‐porphyrin MOF PCN–224(Fe)–CO, which contains heme‐like centers at which photolysis of the Fe‐CO bond can be generated by a femtosecond laser pulse. This is also the first example of an smSFX study performed on a fixed target grid, which the authors highlight offers benefits for chemical systems. These include minimizing sample wastage, which can be particularly important for crystalline solids containing high‐value metal (e.g., Zr) and ligand (e.g., iron tetrakis(4‐carboxylatophenyl)porphyrin) components) and eliminating the problem of chemical incompatibility with the carrier fluids that are required for liquid jetting. Although PCN–224(Fe)–CO is not discussed here as specifically displaying radiation sensitivity, many MOFs can display susceptibility to X‐rays depending on their metal and organic linker composition and the identity and proportion of solvent contained in their pores. Furthermore, in the context of this time‐resolved dynamic photocrystallography study, there are significant benefits to performing the experiment in a serial manner. As highlighted by the authors, the optical laser pulse is not particularly effective at penetrating the crystal bulk in small molecule systems, thus SFX approaches allow the use of a high laser power for the single‐shot pump‐probe approach, with no need to be concerned over any potential photobleaching effects. In their study, the authors successfully show that fixed‐target smSFX can be used to study the ultrafast photodynamics in MOF microcrystals, delivering crystal structure data of high enough quality to elucidate the mechanisms of CO photolysis at picosecond resolution, including the identification of significant difference electron density in the photodifference maps that are used to rationalize the motion of both the dissociated CO and the wider MOF lattice at various time points along the reaction pathway (Figure 9).

A key factor in the success of this study is the use of a comparatively hard X‐ray energy at 14.5 keV, which was provided at the Pohang Accelerator Laboratory X‐ray free‐electron laser (PAL‐XFEL). Access to higher energy (harder) X‐ray wavelengths is not typical at all existing XFEL facilities, which have primarily been developed for the study of biological samples and the use of other X‐ray techniques for which softer X‐rays are more suitable. Pushing the X‐ray energy to 14.5 keV results in a contracted diffraction pattern due to the reciprocal relationship between X‐ray energy and the Bragg peak spacings in the diffraction pattern. Thus, the authors were able to maximize the number of Bragg peaks recorded on the detector for each individual crystal, allowing ab initio indexation of the unit cell parameters from each image in the same manner as achieved for a protein crystal, without the need for other sparse indexing solutions. This highlights the importance of considering the XFEL machine parameters when choosing the best facility to perform smSFX work. As new XFEL projects around the world are conceptualized, it is therefore important that structural chemistry researchers make themselves part of the conversation about the planned machine specifications, as these will necessarily dictate the availability of future facilities at which the smSFX discipline can continue to grow.

#### 3.2.2 Multi‐Crystal Methods at Synchrotrons

As outlined in Section 2, interest in smSX methods is now growing at synchrotron sources as upgrade projects deliver unprecedented increases in X‐ray brilliance, opening the door to further exciting and innovative structural studies but also the potential for increased X‐ray damage. Only a handful of studies have been reported on microcrystalline chemical species to‐date, which are discussed here.

**Figure 10:**
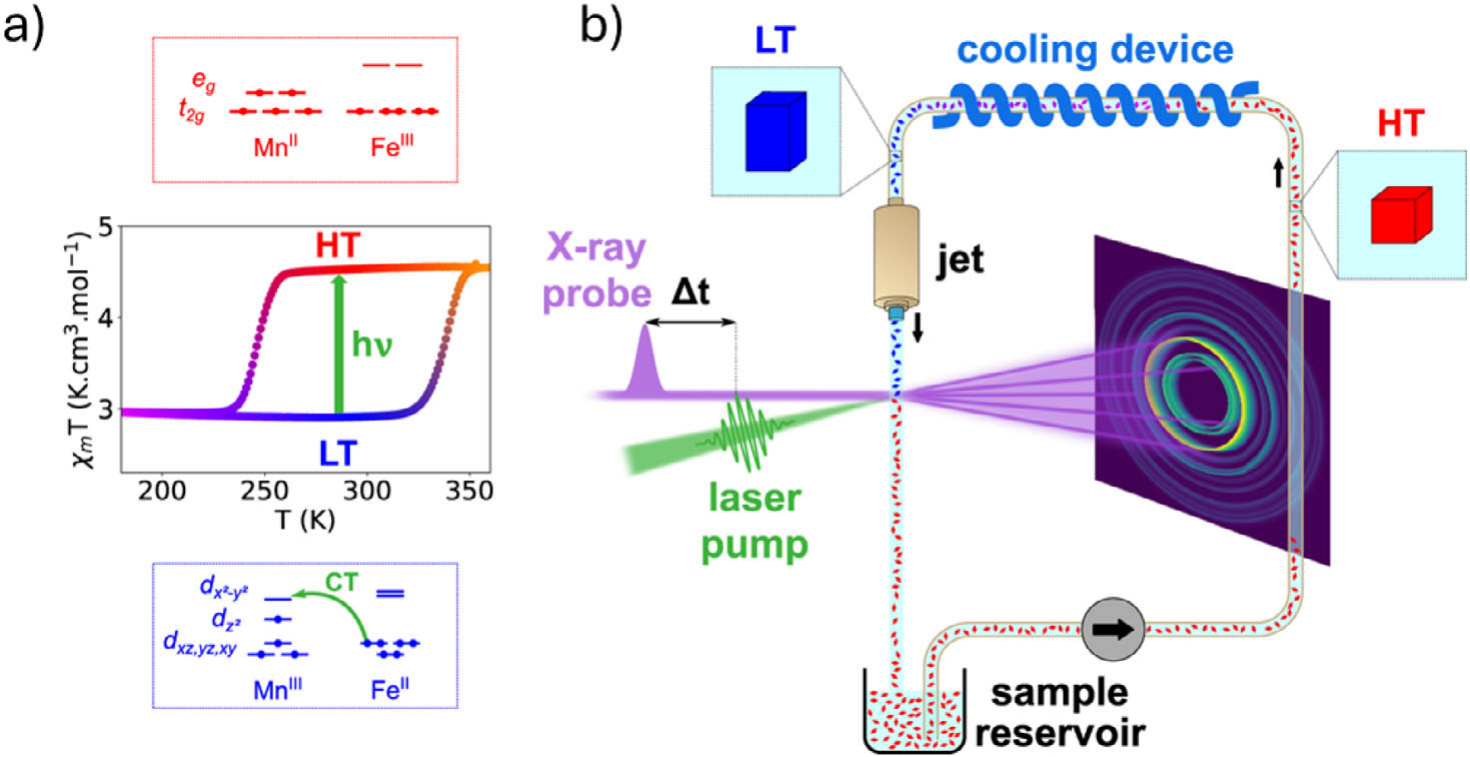
Details of the time‐resolved powder streaming approach developed by Collet and coworkers: a) diagram of the thermal hysteresis between the HT and LT phases of Rb_0.94_Co_0.06_Mn_0.94_[Fe(CN)6]_0.98_·0.2H_2_O along with a schematic representation of the electronic configurations associated with the two phases, and b) an illustration of the streaming powder diffraction technique used for time‐resolved measurements, which shows the closed‐loop circulation system that cools the photoirradiated sample back to the LT phase before reinjection. [Reprinted with permission from Hervé et al*., Nature Communications* 2024, 15, 267, under a Creative Commons Attribution 4.0 International License:  http://creativecommons.org/licenses/by/4.0/. Copyright 2024 Springer Nature.]^[^ ^76^ ^].^

In January 2024, Collet et al. reported a time‐resolved PXRD study performed on beamline ID09 at the ESRF. Their study involved the use of a liquid jet system to deliver a constant stream of sub‐micrometer‐sized crystals (0.9 ± 0.3 µm) into the synchrotron beam, matched to a pulsed laser pump to collect picosecond‐resolved powder diffraction patterns (Figure 10).^[^ ^76^ ^]^ The authors used this time‐resolved powder streaming approach to follow the structural dynamics associated with out‐of‐equilibrium photoswitching inside the thermal hysteresis for the PBA Rb_0.94_Co_0.06_Mn_0.94_[Fe(CN)_6_]_0.98_·0.2H_2_O. Here, excitation by a single laser pulse induced irreversible Mn^III^Fe^II^ → Mn^II^Fe^III^ charge transfer alongside a permanent phase transition from tetragonal to cubic symmetry. This serial powder streaming approach provided a time resolution *Δt* of ca. 35 ps, allowing elucidation of the key steps in the photoinduced phase transition mechanism. Interestingly, the material response was found to be markedly different when excited at different laser fluences. At lower fluence levels (15 mJ cm^−2^), Rietveld refinements showed a small anisotropic unit cell expansion occurring over the first 100 ps that gradually relaxed back to the ground state over a period of 10 µs. These changes were attributed to only a small (ca. 10%) population of the photoinduced cubic phase. A higher laser fluence of 115 mJ cm^−1^ produced a more sudden and significant change, with the unit cell lengths converging toward a single value to reflect a global tetragonal to cubic symmetry change, accompanied by a much larger expansion of the unit cell. Crucially, this first smSSX study highlights another important use for serial approaches, in allowing the determination of fast structural dynamics for irreversible phenomena. As the authors highlight, this experiment could not have been performed on a single crystal by traditional stroboscopic pump‐probe diffraction methods, as these necessitate a reversible crystal switch.

Collet et al. comment that they were limited to using PXRD methods in their study, as the small unit cell parameters and high symmetry of Rb_0.94_Co_0.06_Mn_0.94_[Fe(CN)_6_]_0.98_·0.2H_2_O (*a *= *b* = 7.0747(8) Å and *c *= 10.4744(16) Å in the tetragonal phase) limit the number of Bragg peaks recorded in a single image. This resonates strongly with the challenges expressed for smSFX in the previous section. Similar to the work of Ihee et al. in their SFX breakthrough at the PAL‐XFEL,^[^ ^75^ ^]^ the next advance for smSSX was made in the study of an MOF material by Shepard et al. using a fixed‐target SX methodology on the PROXIMA 2A beamline at Synchrotron SOLEIL.^[^ ^77^ ^]^ In September 2024, they reported the structure determination of MIL‐100(Fe) from microcrystal batches in two different size ranges (10–30 and 1–5 µm). MIL‐100(Fe) presented a suitable step forward in the development of smSSX protocols as, although it presents with a comparatively large unit cell for a chemical crystal system, its high‐symmetry cubic unit cell (*Fd*3*m*, *a* = 73.09(18) Å in this study) still results in a considerably sparse diffraction pattern compared to those typically studied in protein SSX, due to a large number of systematically absent Bragg peaks. The authors present two separate smSSX strategies that are benchmarked on MIL‐100(Fe): one using a true “single shot” SX collection strategy as performed for SFX and another implementing a small rotation at each grid position of between 20 and 60°. During the rotation, a series of data frames were recorded at each grid position in a manner similar to the angular rotation (e.g., an ω‐ or φ‐scan) approach used in standard SCXRD data collections. As the crystal structure of MIL‐100(Fe) was previously known, the authors were able to use the known parameters to support unit cell indexing; however, they also confirmed it was possible to determine the cell parameters ab initio in several instances. The approach was further benchmarked against the Zn(II)‐based MOF ZIF‐8, which displays a considerably smaller, though still moderately large, cubic unit cell with *a* = 16.99(4) Å. Overall, this work represents a considerable step forward in the study of chemical framework microcrystals by smSSX. This is significant given the inherent challenges of MOF diffraction experiments, where the crystals formed are typically small and often only weakly diffracting due to the incorporation of solvent molecules within the pores. SSX approaches provide the option to utilize all of the available X‐ray flux from the synchrotron beam to maximize the diffraction intensity from MOF crystals, without the need to protect the sample from accumulated X‐ray damage. This is a benefit that will only become more significant in the near future, as facilities continue to upgrade their capabilities.

**Figure 11:**
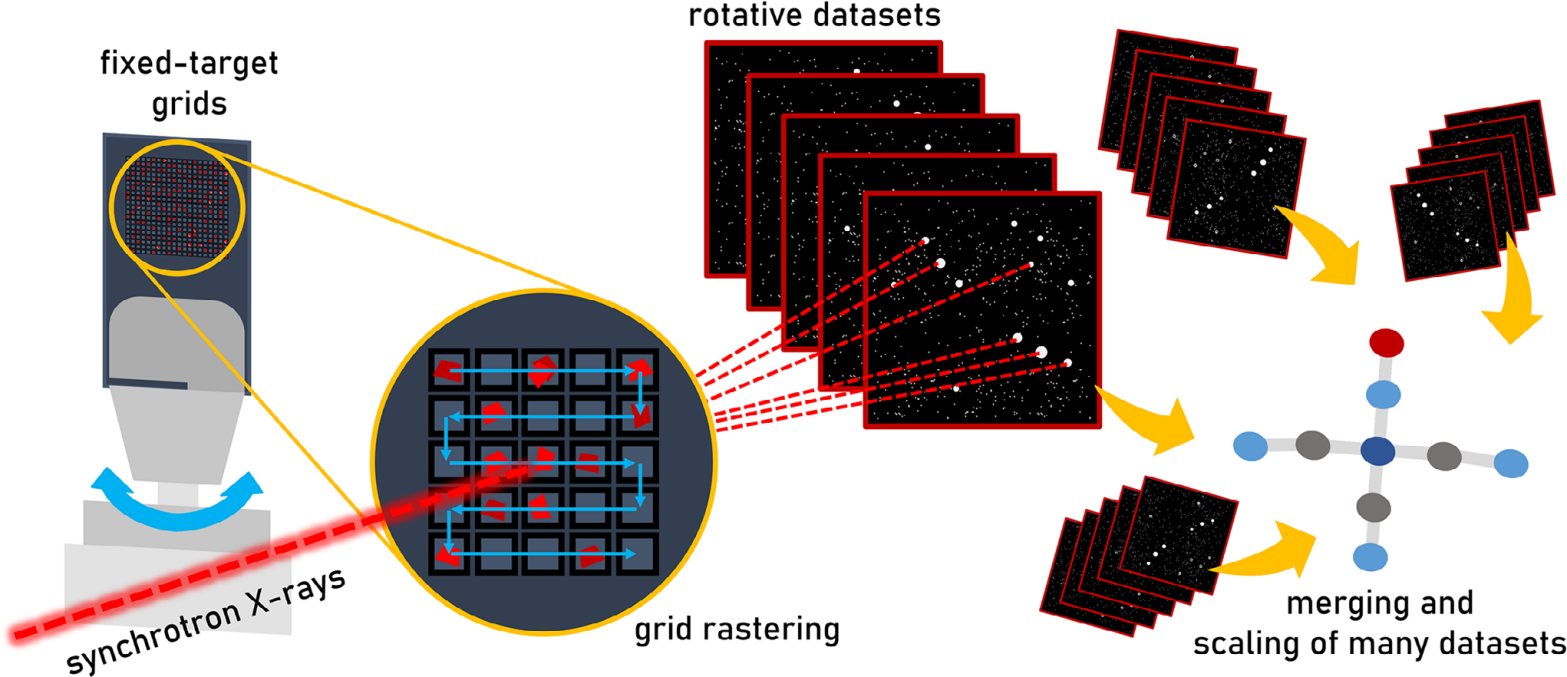
Schematic summary of the small‐rotative fixed‐target serial synchrotron crystallography (SR‐FT‐SSX) technique developed by Lewis et al. for serial crystal structure determination of small molecule crystal structures at the UK synchrotron DLS.^[^ ^78^ ^].^

In parallel with the work of Shepard et al., the current authors have been working to develop smSSX methods on beamline I19 at the UK synchrotron facility, DLS. In December 2024, we reported a small‐rotative fixed‐target SSX (SR‐FT‐SSX) data collection and processing pipeline for the study of small molecule crystal systems with very small unit cells.^[^ ^78^ ^]^ Our approach was benchmarked against the Fe(II) compound sodium nitroprusside dihydrate (SNP.2H_2_O), which has unit cell parameters in the range of 6–16 Å and displays orthorhombic *Pnnm* symmetry, resulting in several systematically absent Bragg peaks. In a similar manner to Shepard et al., the SR‐FT‐SSX methodology implements a small rotation about the diffractometer φ‐axis to generate a short series of diffraction images at each fixed‐target grid position, which are then treated as a partial dataset in the subsequent processing pipeline (Figure 11). Using SSX processing protocols in the program DIALS,^[^ ^79^ ^]^ we are able to index the data from each grid position to determine the unit cell parameters and orientation matrix for each microcrystal ab initio from every partial dataset, which are then merged and scaled for structure solution of the final serial crystal structure using standard methods. During data collection, we performed rotational scans of 5° to deliver 50 diffraction images in each partial dataset for unit cell indexing, which, despite being a considerably smaller rotation than that used to study the MOF crystals in the previous example, still provided crystal structure data that was of comparable, or in some cases better quality than comparative SCXRD datasets. Further data processing iterations, using progressively fewer images, confirmed that ab initio unit cell indexing can be achieved with as little as a 0.3° rotation (i.e., collection of only 3 × 0.1°‐wide φ‐scan images), which indicates the exciting possibility to use SR‐FT‐SSX to study crystals that are severely sensitive to the X‐ray beam. Another key strength of the method is the design of on‐the‐fly data processing regimes and the generation of spatially resolved plots for fast result visualization across the fixed‐target grid. The tools provide real‐time analysis of the smSSX data quality that can facilitate fast decision‐making for the experimenter, maximizing the use of precious synchrotron beamtime.

As is evident from the developments discussed, which have all been published in a 12‐month period, smSSX is a nascent area of X‐ray diffraction research. What is obvious across the developments in SFX and SSX on small molecule crystals is the fact that this research is a global endeavor that is capitalizing on the wealth of state‐of‐the‐art research facilities available to crystallographic experimenters around the world. The authors are aware of a number of new smSX reports that are currently in press, and thus we anticipate that the discipline will continue on its current upward trajectory for many years to come.

## 4 Summary and Outlook

In summary, it is clear from the depth and breadth of the research explored across this review that X‐ray‐induced processes are prevalent in chemical crystalline materials, despite a long‐held view that radiation damage is far less of a problem for small molecule than for MX. The rising number of recent reports exploring both X‐ray damage and other radiation‐induced processes in small molecule crystals and microcrystalline powders reflects renewed interest in the area and is intuitively the result of the dramatic and continuing advances in X‐ray generation technologies being enjoyed by crystallographic disciplines around the world. In all cases, quantification is key, and protocols to allow the estimation of the accumulated dose in small molecule crystals are now readily available. We note that, while dose calculations, where possible, provide a route to quantify the X‐ray exposure levels received by a sample, many of the studies discussed in this article showcase that the structural response to radiation damage *can* be elucidated using diffraction measurements, providing the overall exposure level is kept low. Understanding X‐ray‐induced effects is particularly important for in situ studies of functional materials, including in light‐pump‐X‐ray‐probe studies where prior confirmation of the impact of the probe X‐ray beam is essential to allow reliable conclusions to be drawn as to the impact of other external stimuli on the sample. These conclusions highlight the need to consider the potential for X‐ray‐induced effects in all experiments, particularly those being performed using state‐of‐the‐art X‐ray sources at accelerator facilities, and, where necessary, to consider the most appropriate methods to address the excessive accumulation of X‐ray dose. The required mitigation approach needn't always be the most complicated, and straightforward solutions involving readily controlled parameters such as temperature, exposure time, and attenuation should always be considered as a first step. Where these measures are proven to be insufficient, innovative approaches such as multi‐sampling and multi‐crystal data collection methodologies are now developing at pace for both synchrotron and XFEL studies. As smSFX and smSSX methods become more established, they should now be stress‐tested against a much broader range of functional chemical materials and problems. In particular, pioneering research into in situ and dynamic SFX studies offers a tantaslizing glimpse of the potential for time‐resolved SX to provide real‐time mechanistic information on a range of dynamic solid‐state processes, insight with the power to revolutionize our understanding of solid‐state chemistry on the picosecond to femtosecond timescale.

Finally, recent literature highlights a shift in perspective: that X‐ray‐induced effects in crystalline materials are not merely problematic but can also present valuable opportunities for discovery and methodological advancement. A number of challenges remain to unlock the potential of X‐ray excitation in chemical crystalline materials, most particularly the fact that the physical basis for X‐ray interaction with the sample is not yet established for many systems. Comprehensive work by Fernando et al.^[^ ^53a^ ^]^ highlights the key role of complementary techniques in providing a comprehensive picture of the complete response to X‐rays at the crystal, chemical, and electronic scale, and this study should serve as an inspiration for others to fully investigate the interesting X‐ray‐induced processes occurring in their samples. The ability to harness these effects offers the exciting prospect of radiation‐induced control in the crystalline state, transforming X‐rays from a problematic, noninnocent probe into a powerful new route to manipulate and tune material properties.

## Conflict of Interest

The authors declare no conflict of interest.

## Data Availability

Data sharing is not applicable to this article as no new data were created or analyzed in this study.
